# Thorasic Abscess—Retained Placenta after Abortion

**Published:** 1873-11-01

**Authors:** J. Schneck

**Affiliations:** Mt. Carmel, Ill.


### THORASIC ABSCESS—RETAINED PLACENTA AFTER ABORTION.

NOTES OF TWO CASES FROM PRACTICE, BY

J. SCHNECK, M.D., MT. CARMEL, ILL.

CASE I.—*Thorasic Abscess.*—Was called December 10th, 1871, to see E. C——, a robust boy of 13 years; whom I found to have pneumonia, involving all of the left lung and pleura, also, to some extent, the lower half of the right lung, which run an unusually obstinate course, until the 19th, when my patient seemed to be safe and convalescing. Heard no more of him until January 9th following, when I found him very much exhausted and emaciated, with a hard swelling situated near midway between the axilla and sternum of the left breast, very tender, and of a dark or purplish color. Thinking it to be an external abscess, ordered it to be poulticed freely, the patient to take a tonic of iron, zinc, cinchonia, and columbo; with directions to send me word when there was any softening or pointing.

January 23d was again called; found my patient hectic, and nervous system very excitable; the tumor was now soft, and involved the greater part of the left precordia, the surface of which was raised near two inches at the highest point, higher than on the right side; and pulsating very strongly at each beat of the heart.

January 24th, after consultation with Dr. Rigg, it was decided to open the abscess. The knife was followed by as large a stream of pus as the opening would permit, of a yellowish green color, passing at first some five feet from the patient, which distance was increased a foot or more at each beat of the heart.

This was allowed to run, until about five pints had been passed. A tent was then placed in the opening.

January 25th, the tent was removed, and near another pint of pus came away. The cavity was now injected with a weak solution of carbolic acid and water; this treatment was continued twice a day, with the above tonics, until at the end of three weeks, when the cavity was healed up; and in three months my patient was stout and rugged as ever.

Case II.—*Retained Placenta following Abortion.*—October 19th was called to see Miss A. C——, aged 20 years, some larger than the average, both in height and general development. On my way out (she lived four miles in the country) her paramour gave me the following history:

That she had not menstruated for near five months. That she had taken two boxes of "Cherokee Pills," but with no effect. Had given her various kinds of injections, among the rest a solution of "blue vitriol," but all to no effect. That five days since, October 14th, he had succeeded in "breaking the waters with a bent piece of wire," and that she had been losing blood ever since.

Upon my arrival I found her looking very anxious and pale. Tongue moist, with a white fur coating; pulse small, but quick; abdomen somewhat swollen and tender; with a copious and sanious discharge per vaginum; by the touch was revealed a soft, spongy substance in the cervix uteri.

She informed me, that on the night of the 15th she suffered very severe pain, and during the night passed a "lump of something about as large as a rat," and since that time had been passing more or less blood, and suffering much from pain.

Regarding the case as one of retained placenta, and being strenuously refused the privilege of taking it away, there was no alternative left but to give ergot, and wait the result. Heard no more of my patient until the 23d, when I was sent for in great haste. This time found her in a toxemic condition; delirious; tongue dry in the center, and heavily coated; pulse very rapid, but the heat of surface not much increased, except the abdomen, which was still swollen and hot. I now at once proceeded to remove the decaying and excessively fœtid afterbirth; this accomplished, I introduced into the womb a No. 12 catheter, to the free end of which I fixed a syringe; with this arrangement I was enabled to give the inner surface of the uterus a thorough washing-out, which I did twice this day. Also ordered forty drops tinct. ferri, perchlor., every three hours, to which had been added as much potass. chloras, as it would dissolve, and alternate with this six grains quinia sulph.

The next day found my patient some improved. The interval between the doses was lengthened, and the dose lessened, as convalescence gradually came on. By the 28th I dismissed the patient cured.

I regard the above cases as of interest:

1. By showing the beneficial effect of carbolic acid; the strength used in each case was about five per cent.

2. The decided anti-toxemic effect of tinct. ferri, perchlor., and quinia sulph., of which I hope in future to have more to say.

3. The unmolested way in which murderers are allowed to advertise their business. The above pills were obtained through an advertisement found in one of the leading St. Louis weekly papers, at a cost of $8.00, with directions that, if the pills did not accomplish the object, to send the *victim* to St. Louis, to their "Home for the Sick." Is it not time that those who sit in high places should look after this? Nor is this an isolated case. All you have to do is to carefully read the paper that daily comes to your office and family circle, to find the same character of advertisements.

---

British Association for the Advancement of Science.—The meeting this year has been held at Bradford, and many interesting and highly scientific papers were read. In consequence of the illness of Dr. Joule, the Presidential Chair was occupied by Professor Williamson, who delivered an address that is exciting much attention.

