# Correction

**Published:** 1873-11-15

**Authors:** 


CORRECTION.—In article, "Thorasic Abscess," on page 245 of our last number, in line seventeen, "zinc" should read tinct.

